# Supplementary material for: Lack of Evidence for the Role of the p.(Ser96Ala) Polymorphism in Histidine-Rich Calcium Binding Protein as a Secondary Hit in Cardiomyopathies
Source: Int J Mol Sci. 2023 Nov 3;24(21):15931. doi: 10.3390/ijms242115931 (PMC10648441; doi:10.3390/ijms242115931)
Supplement: Supplementary file 1 [file ijms-24-15931-s001.zip › Table S1.pdf]

Supplementary Materials

Table S1. Logistic regression analysis in *PLN* p.(Arg14del) index or relatives were modelled with *HRC* Ser96Ala as predictor. Analyses were adjusted for sex, age at enrollment and principal components. PLN; phospholamban, CI; confidence interval, VA; ventricular arrhythmia.

***PLN* p.(Arg14del)**	**Odds ratio**	**95% CI**	***p*-value**
	**Index-only**		
**Life-threatening VA event**	0.554	0.300 – 0.985	0.049*
**HF event**	0.887	0.509 – 1.535	0.667
**Composite**	0.655	0.376 – 1.120	0.126
	**Relative-only**		
**Life-threatening VA event**	0.994	0.627 –1.561	0.981
**HF event**	0.885	0.480 – 1.606	0.690
**Composite**	1.024	0.681 – 1.528	0.908

*after correction for multiple testing, this statistical analysis was not sustained.

Table S1a. Time-to-event analyses in *PLN* p.Arg14del carriers. PLN; phospholamban, PC; principal component, CI; confidence interval, Ser; serine, Ala; alanine, VT; ventricular tachycardia, HF; heart failure.

PLN* *p.(Arg14del)	**Adjusted for sex, age at enrollment and PC**				
	Estimate	Standard error	Hazard ratio	95% CI of Hazard ratio	*p*-value
**Genetic variant Ser96Ala T > G**					
**Incident death (all cause mortality) (from birth)**	-0.127	0.188	0.880	[0.609 – 1.273]	0.499
**Incident death (all cause mortality) (from enrollment)**	-0.176	0.183	0.839	[0.5856– 1.202]	0.338
**Incident VT/VF (from birth)**	-0.206	0.139	0.814	[0.620 – 1.068]	0.137
**Incident VT /VF (from enrollment)**	-0.209	0.137	0.812	[0.621 – 1.061]	0.126
**Incident HF (from birth)**	-0.134	0.151	0.874	[0.651 – 1.174]	0.373
**Incident HF (from enrollment)**	-0.111	0.148	0.895	[0.670 – 1.196]	0.453
**Incident composite (from birth)**	-0.130	0.116	0.878	[0.700 – 1.103]	0.264
**Incident composite (from enrollment)**	-0.136	0.114	0.873	[0.698 – 1.093]	0.236

Table S1b. Time-to-event analyses in the DCM cohort. DCM; dilated cardiomyopathy, PC; principal component, CI; confidence interval, Ser; serine, Ala; alanine, VT; ventricular tachycardia, SCA; sudden cardiac arrest, ICD; implantable cardioverter defibrillator.

DCM	Adjusted for sex, age at enrollment, genotype array and PC				
	Estimate	Standard error	Hazard ratio	95% CI of Hazard ratio	*p*-value
**Genetic variant Ser96Ala T > G**					
Incident death (from enrollment)	0.131	0.097	1.140	[0.94 – 1.38]	0.172
Incident death (from DCM diagnosis)	0.131	0.097	1.140	[0.94 – 1.38]	0.176
Incident VT (from enrollment)	-0.086	0.141	0.918	[0.70 – 1.21]	0.543
Incident VT (from DCM diagnosis)	-0.092	0.141	0.912	[0.69 – 1.20]	0.516
Incident SCA (from enrollment)	0.141	0.222	1.152	[0.75 – 1.78]	0.525
Incident SCA (from DCM diagnosis)	0.123	0.221	1.131	[0.73 – 1.74]	0.579
ICD implantation (from enrollment)	-0.054	0.099	0.947	[0.78 – 1.15]	0.585
ICD implantation (from DCM diagnosis)	-0.061	0.099	0.941	[0.77 – 1.14]	0.541
