# Supplementary material for: Lack of Evidence for the Role of the p.(Ser96Ala) Polymorphism in Histidine-Rich Calcium Binding Protein as a Secondary Hit in Cardiomyopathies
Source: Int J Mol Sci. 2023 Nov 3;24(21):15931. doi: 10.3390/ijms242115931 (PMC10648441; doi:10.3390/ijms242115931)
Supplement: Supplementary file 1 [file ijms-24-15931-s001.zip › Table S2.pdf]

Table S2

Table S2. Time-to-event analyses in *PLN* p.Arg14del carriers were modelled with *HRC* Ser96Ala as predictor. Analyses were adjusted for sex, age at enrollment and principal components. PLN; phospholamban, CI; confidence interval, VA; ventricular arrhythmia, HF; heart failure.

PLN p.(Arg14del)	**Hazard ratio**	**95% CI of Hazard ratio**	**p-value**
**Incident death (all cause mortality) (from birth)**	0.880	[0.609 – 1.273]	0.499
**Incident death (all cause mortality) (from enrollment)**	0.839	[0.5856– 1.202]	0.338
**Incident life-threatening VA (from birth)**	0.814	[0.620 – 1.068]	0.137
**Incident life-threatening VA (from enrollment)**	0.812	[0.621 – 1.061]	0.126
**Incident HF (from birth)**	0.874	[0.651 – 1.174]	0.373
**Incident HF (from enrollment)**	0.895	[0.670 – 1.196]	0.453
**Incident composite (from birth)**	0.878	[0.700 – 1.103]	0.264
**Incident composite (from enrollment)**	0.873	[0.698 – 1.093]	0.236
