# Supplementary material for: Lack of Evidence for the Role of the p.(Ser96Ala) Polymorphism in Histidine-Rich Calcium Binding Protein as a Secondary Hit in Cardiomyopathies
Source: Int J Mol Sci. 2023 Nov 3;24(21):15931. doi: 10.3390/ijms242115931 (PMC10648441; doi:10.3390/ijms242115931)
Supplement: Supplementary file 1 [file ijms-24-15931-s001.zip › Table S3.pdf]

Table S3

Table S3. Time-to-event analyses in DCM patients were modelled with *HRC* Ser96Ala as predictor**.** DCM; dilated cardiomyopathy, CI; confidence interval, VT; ventricular tachycardia, SCD; sudden cardiac death, ICD; implantable cardioverter defibrillator.

DCM	**Hazard ratio**	**95% CI of Hazard ratio**	**p-value**
**Incident death (from enrollment)**	1.140	[0.94 – 1.38]	0.172
**Incident death (from DCM diagnosis)**	1.140	[0.94 – 1.38]	0.176
**Incident VT (from enrollment)**	0.918	[0.70 – 1.21]	0.543
**Incident VT (from DCM diagnosis)**	0.912	[0.69 – 1.20]	0.516
**Incident SCD (from enrollment)**	1.152	[0.75 – 1.78]	0.525
**Incident SCD (from DCM diagnosis)**	1.131	[0.73 – 1.74]	0.579
**ICD implantation (from enrollment)**	0.947	[0.78 – 1.15]	0.585
**ICD implantation (from DCM diagnosis)**	0.941	[0.77 – 1.14]	0.541
